# Dietary Intake and Status of Vitamin B12 in Slovenian Population

**DOI:** 10.3390/nu14020334

**Published:** 2022-01-13

**Authors:** Živa Lavriša, Hristo Hristov, Maša Hribar, Katja Žmitek, Anita Kušar, Barbara Koroušić Seljak, Matej Gregorič, Urška Blaznik, Nadan Gregorič, Katja Zaletel, Adrijana Oblak, Joško Osredkar, Igor Pravst

**Affiliations:** 1 Nutrition Institute, Tržaška Cesta 40, SI-1000 Ljubljana, Slovenia; ziva.lavrisa@nutris.org (Ž.L.); hristo.hristov@nutris.org (H.H.); masa.hribar@nutris.org (M.H.); katja.zmitek@vist.si (K.Ž.); anita.kusar@nutris.org (A.K.); 2 Biotechnical Faculty, University of Ljubljana, Jamnikarjeva 101, SI-1000 Ljubljana, Slovenia; 3 VIST–Faculty of Applied Sciences, Gerbičeva Cesta 51A, SI-1000 Ljubljana, Slovenia; 4 Computer Systems Department, Jozef Stefan Institute, SI-1000 Ljubljana, Slovenia; barbara.korousic@ijs.si; 5 National Institute of Public Health, Trubarjeva 2, SI-1000 Ljubljana, Slovenia; matej.gregoric@nijz.si (M.G.); urska.blaznik@nijz.si (U.B.); 6 University Medical Centre Ljubljana, Zaloška Cesta 7, SI-1000 Ljubljana, Slovenia; nadan.gregoric@kclj.si (N.G.); katja.zaletel@kclj.si (K.Z.); adrijana.oblak@kclj.si (A.O.); josko.osredkar@kclj.si (J.O.); 7 Faculty of Medicine, University of Ljubljana, Vrazov trg 2, SI-1000 Ljubljana, Slovenia; 8 Faculty of Pharmacy, University of Ljubljana, Aškerčeva Cesta 7, SI-1000 Ljubljana, Slovenia

**Keywords:** vitamin B12, deficiency, homocysteine, folate, Slovenia, EU Menu

## Abstract

Vitamin B12 deficiency poses a health concern, especially in vulnerable populations. Dietary vitamin B12 intake was obtained by two 24 h dietary recalls and food propensity questionnaires in a representative Slovenian cross-sectional food consumption survey, SI.Menu (*n* = 1248 subjects; 10–74 years). For a subgroup of 280 participants, data on serum vitamin B12 were available through the Nutrihealth study. The estimated usual population-weighted mean daily vitamin B12 intakes were 6.2 µg (adults), 5.4 µg (adolescents), and 5.0 µg (elderly). Lower intakes were observed in females. Inadequate daily vitamin B12 intake (<4 µg) was detected in 37.3% of adolescents, 31.7% of adults, and 58.3% elderlies. The significant predictors for inadequate daily vitamin B12 intake were physical activity score in all age groups, sex in adolescents and adults, financial status and smoking in elderly, and employment in adults. Meat (products), followed by milk (products), made the highest vitamin B12 contribution in all age groups. In adolescents, another important vitamin B12 contributor was cereals. The mean population-weighted serum vitamin B12 levels were 322.1 pmol/L (adults) and 287.3 pmol/L (elderly). Low serum vitamin B12 concentration (<148 nmol/L) and high serum homocysteine (>15 µmol/L) were used as criteria for vitamin B12 deficiency. The highest deficiency prevalence was found in elderlies (7.0%), particularly in males (7.9%). Factors associated with high serum homocysteine were also investigated. In conclusion, although vitamin B12 status was generally not critical, additional attention should be focused particularly to the elderly.

## 1. Introduction

Vitamin B12, also known as cobalamin, is a water-soluble vitamin that plays a significant role in cellular metabolism. It acts as a cofactor in metabolic processes that are important, especially in DNA methylation and synthesis, as well as in mitochondrial metabolism [1]. Consequently, it is required for the normal functioning of the nervous system and red blood cell formation. Vitamin B12 deficiency can cause megaloblastic anemia, where, due to its incapacity for DNA synthesis, the bone marrow produces large blood cells with immature nuclei [2]. Other prominent manifestations of B12 deficiency are neuropsychiatric, with a wide range of symptoms, whereby the elderly are particularly at risk [3,4]. The metabolism of vitamin B12 is closely intertwined with folate [5]; insufficient vitamin B12 status can also lead to a functional folate deficiency, as folate becomes trapped and cannot be properly utilized [6]. Disruption of these metabolic pathways can lead to the accumulation of homocysteine, which is a known risk factor for cardiovascular diseases, such as atherosclerosis, endothelial dysfunction, and thromboembolism [7,8,9,10].

Naturally, vitamin B12 is produced by bacterial synthesis [11]. Its main foods sources include foods of animal origin, such as meat, fish, eggs, and dairy [12]. Foods of plant origin are typically not sources of this vitamin; however, certain fermented foods, such as fermented soybean (tempeh), can contain considerable amounts of vitamin B12, depending on the production procedure [13]. It should be also noted that blue-green algae (cyanobacteria), which can be found in some dietary supplements, usually contain pseudovitamin B12, which is inactive in humans [12]. A valuable source of vitamin B12 for those who avoid foods of animal origin can be fortified foods, i.e., breakfast cereals. In the European Union (EU), food fortification with vitamin B12 is voluntary, with cyanocobalamin and hydroxocobalamin as authorized sources [14]. Vitamin B12 naturally present in foods is bound to peptides, proteins, and glycoproteins, and must be released during the digestion process to be utilized by the body. The bioavailability of vitamin B12 is the greatest in fish, meat, and milk (42–89%) [12,15] and lower in eggs (<9%) [16]. It should be acknowledged that at high single doses of vitamin B12, the proportion of absorbed vitamin B12 declines, so dispersing the intake among daily meals could result in a higher amount being absorbed [17,18]. Losses in vitamin B12 in foods can predominantly occur during storage, and with light and heat exposure, with losses between 30% and 50% [12,19]. The presence of other vitamins can also affect its stability [20].

The major causes of vitamin B12 deficiency are inadequate intake, inadequate bioavailability, and malabsorption. An inadequate intake of vitamin B12 can occur especially in subjects who exclude foods of animal origin from their diet, such as vegans [21,22], but is present in the general population as well. Low dietary intake usually does not result in symptomatic cobalamin deficiency until hepatic reserves are exhausted [23]. In the elderly, the main risk for vitamin B12 deficiency is usually due to impaired absorption, and less due to inadequate dietary intake [24,25,26]. Food-cobalamin malabsorption, which is primarily caused by gastric atrophy, is a common cause of vitamin B12 deficiency, particularly in the elderly, and is often also caused by infection with *Helicobacter pylori* [23]. Another common risk factor for developing B12 deficiency due to malabsorption is pernicious anemia, in which a lack of gastric intrinsic factor impairs the intestinal B12 absorption [27]. Vitamin B12 deficiency and anemia can also occur in some long-term drug treatments, such as metformin in diabetic patients, so for these patients, routine testing for deficiency and/or supplementation is advised [28]. Additionally, other risk factors for deficiency include several gastric diseases and/or surgeries that compromise vitamin B12 absorption [1]. Several diagnostic approaches are available for the diagnosis of vitamin B12 malabsorption and deficiency [29], and a food-cobalamin malabsorption test can be used to determine the organism’s ability to release vitamin B12 from foods [30].

To evaluate serum vitamin B12 status, several biomarkers exist. For a general estimation of vitamin B12 status, total serum vitamin B12 concentration is typically used, which measures both holotranscobalamin and transcobalamin [31]. Additionally, holotranscobalamin (bioactive protein-bound form of vitamin B12) can be used as a marker of vitamin B12 status, together with methylmalonic acid (MMA) and homocysteine (HC) to test for deficiency [32]. These auxiliary markers are used because the serum vitamin B12 alone has limited diagnostic value. While currently there is no consensus on the best marker or best combination of markers to be applied for the assessment of vitamin B12 status, it is advised to use at least one metabolic biomarker together with serum vitamin B12 levels [33]. In the literature, vitamin B12 serum status in adults is commonly defined with the following cut-offs: >221 pmol/L—vitamin “B12 adequacy”, 148–221 pmol/L—“low B12”, and <148 pmol/L—“B12 deficiency” [33,34,35,36]. Homocysteine concentrations above 15 μmol/L are typical for vitamin B12 deficiency [35,37]. In the present study, homocysteine concentrations between 10 and 15 μmol/L were considered as marginally elevated. As a criterion for high-risk vitamin B12 deficiency, serum vitamin B12 concentration <148 pmol/L, together with homocysteine concentration >15 μmol/L, was applied.

Internationally, there are quite considerable differences in reference intake values for vitamin B12. The World Health Organization (WHO) and the United States Institute of Medicine (IoM) reference value for vitamin B12 intake is set at 2.4 µg/day for adolescents and adults [38]. Nordic Council of Ministers’ reference value is even lower, at 2 µg for adolescents and adults [39]. The current reference daily vitamin B12 intake in Slovenia [40], which was implemented according to German–Austrian–Switzerland (D-A-CH) recommendations [41], is 3.5 µg for adolescents of 10–13 years and 4 µg for adolescents older than 13 years and adults, including the elderly [40]. It should be noted that the D-A-CH reference intake was previously set at 3 µg but was recently revised considering newer vitamin B12-related biomarker studies [42]. The same reference values are also set by the European Food Safety Authority (EFSA) [43].

According to the WHO, in some populations worldwide, vitamin B12 status is a public health concern [34]. However, more country representative data are lacking for assessment [44]. Vitamin B12 deficiency is more prevalent in less developed countries, which is closely related to the diet. In Latin America and some parts of Africa, around 40% of the population is deficient [45], and these proportions are even higher in parts of India, where deficiency prevalence can reach up to 70% of the population. In a recent study from Latin America, particularly elderly women (33%) were found to be vitamin B12 deficient [46]. In developed countries, such as the United States of America and the United Kingdom, deficiency is around 6% in the general population and around 20% in the elderly population [47]. A study on European populations reported serum vitamin B12 concentrations mostly around 350 pmol/L, with few countries reporting marginal vitamin B12 status [48]. Data from dietary surveys in nine European Union countries showed that average vitamin B12 intakes across countries range between 3.3 and 6.6 μg/day in adolescents (10–18 years) and 4.2 and 8.6 μg/day in adults [43]. In Slovenia, comprehensive, nationally representative data on B12 status or intake in the population have not been available; this topic has only been investigated in a few studies on smaller population subgroups or patients [49,50,51,52].

The objective of the present study was to estimate the dietary vitamin B12 intake and prevalence of vitamin B12 deficiency in the Slovenian population. The secondary objective was to determine the main sources of vitamin B12 in the daily diet and the determinants of low vitamin B12 intake and deficiency. Vitamin B12 intake was investigated for adolescents, adults, and the elderly using the data collected in the nationally representative food consumption SI.Menu study, while vitamin B12 status was assessed using Nutrihealth study data on adults and the elderly.

## 2. Material and Methods

### 2.1. Study Design and Participants

Data for the present study were obtained from the nationally representative cross-sectional food consumption study SI.Menu. Data were collected between March 2017 and April 2018 in Slovenia (population ca. 2 million). The study was performed based on the European Food Safety Agency (EFSA) Guidance on EU Menu Methodology [53]. The SI.Menu study methodology details have previously been published [54]. Participants were randomly selected from the Central Register of Population of Slovenia, with consideration of place of residency, age, and sex. Participants were selected only from private households; people living in institutions were not included. Statistical units were individuals. The study sample included 2280 participants, stratified into three age groups: adolescent (10–17 years), adult (18–64 years), and elderly population (65–74 years). The final response rate was 62% (1319 participants); the lowest participation rates were observed in adults (57%), and these were higher in adolescents (69%) and the elderly (65%). In these two groups, participation rates were very similar between males and females, while in the adults, the participation rate of males was notably lower (50% vs. 61%). The sampling was performed according to the EFSA guidelines on minimum sample sizes for each of the age groups [53], with consideration of sample sizes of previous Slovenian dietary surveys [55,56]. The population of Slovenia at the time of the sampling was 2.064 million people. The study protocol was approved by the National Medical Ethics Committee (KME 53/07/16; approval No. 0120-337/2016 issued on 19 July 2016). Prior to inclusion in the study, all subjects were informed about the study and signed an informed consent form. In the case of adolescents, informed consent was also obtained from the parent or legal guardian.

As an extension of the SI.Menu study, data on serum biomarkers were collected via the Nutrihealth study for a subsample of adults and elderly people who participated in a SI.Menu study. Altogether, 34% of adults and 37% of elderly people from the SI.Menu study were further included in the Nutrihealth study. Blood and urine samples were collected for *n* = 280 participants (125 adults and 155 elderly). The sample size is comparable with other previously published studies on this topic [57,58]. In the blood samples, serum vitamin B12 and homocysteine were determined. The detailed methodology of the Nutrihealth study has previously been published [59].

### 2.2. Data Collection

In the SI.Menu study, data were collected using a general questionnaire, a food propensity questionnaire (FPQ), and two 24 h dietary recalls. During the first interview, data from the general questionnaire, anthropometric data, and FPQ data were collected, together with the first 24 h dietary recall. During the second interview, data for the second 24 h dietary recall were collected.

Via the general questionnaire, sociodemographic and socioeconomic data were obtained, such as place of residency, education, employment, financial status, smoking, and the following of specific dietary patterns. A cut-off point for below/above average self-reported financial status was at a monthly income of EUR 1300. Participants also self-reported their level of physical activity, which was transformed into an International Physical Activity Questionnaire (IPAQ) score [60]. Anthropometric data were collected, including body height and body weight for body mass index (BMI) calculation. A cut-off point for overweight was established at 25 kg/m^2^ for adults; those below this cut-off point were grouped into “normal”, even if their BMI was lower than 18.5 kg/m^2^. For adolescents, sex-/age-adjusted cut-off points (>1 standard deviation (SD)) were applied as described in the literature [61,62].

Dietary habits of participants were collected by a trained interviewer performing 24 h dietary recalls for two nonconsecutive days, which were 7–21 days apart, covering workdays and weekends (71% and 29%, respectively). For the estimation of portion sizes, a national, validated picture book was used, which depicted commonly consumed foods and simple dishes in six portion sizes [63]. Further, the usual consumption frequency of specific foods in the past 12 months was collected using FPQ with the following frequency response options: never, 1–3 times per month or less, once per week, 2–3 times per week, 4–6 times per week, 1–2 times per day or more [54].

### 2.3. Dietary Vitamin B12 Intake

Data on food consumption from 24 h recalls were inserted into the Open Platform for Clinical Nutrition (OPEN), which is a web application based on the Slovenian food composition database [64]. The OPEN platform consists of nutritional composition data of branded and generic foods, as well as commonly used recipes in Slovenia. The sample of extracted foods from dietary recalls consisted of 2377 foods. For foods with missing vitamin B12 content in the OPEN, this information was searched for in other food composition databases, particularly the National Food Composition Database in Finland (Fineli) [65] and the United States Department of Agriculture Food Composition Database (USDA) [66]. Altogether, 37.1% of the foods from the sample were found to be sources of vitamin B12. The Multiple Source Method (MSM) was used for estimating usual daily intakes, with consideration of FPQ [67], in line with the previously reported approach [68].

### 2.4. Vitamin B12 and Homocysteine Status

A subsample of adult and elderly participants from the SI.Menu study was included in the Nutrihealth study, where they provided fasting blood samples. Serum vitamin B12 and homocysteine concentrations were measured in human serum at the Department of Nuclear Medicine (University Medical Center, Ljubljana).

Total serum vitamin B12 concentration was analyzed with the chemiluminescence immunoassay, determined on an Immulite 2000 XPi analyzer (Siemens Healthineers, Gwynedd, UK). The performance characteristics of the assay are as follows: the vitamin B12 concentration limit of detection is 92 pmol/L; the linearity of the assay is in the range of 111 to 738 pmol/L with a recovery range of 92% to 123%. The intra-assay and interassay coefficients of variation range from 6.7% to 13.0% and from 6.0% to 15.0%, respectively. The cross-reactivity of the vitamin B12 assay was shown to be nondetectable for cobinamide.

In the assessment of vitamin B12 status, we also considered serum homocysteine levels, already reported in our previous study [68]. A chemiluminescence immunoassay method carried out on an IDS-iSYS analyzer (Immunodiagnostic Systems, Boldon, UK) was used for the determination of homocysteine. A cut-off point for high homocysteine was set at 15 μmol/L [37,69]. In the assessment of factors influencing serum homocysteine levels, we also considered serum folate concentrations, which were previously reported in the above-mentioned paper [68]. Serum folate was measured with the chemiluminescence immunoassay on an Immulite 2000 XPi analyzer (Siemens Healthineers, Gwynedd, UK), and a cut-off of 7 nmol/L was used to identify subjects with low serum folate level.

### 2.5. Data and Statistical Analysis

Data cleaning was conducted separately for each of the two dietary recalls. To examine under- and over-reporting, the adapted Goldberg et al. method was used, as previously described in the study of Black et al. [70]. The method is based on the ratio of reported daily energy intake and basic metabolic rate (BMR). The BMR was estimated using sex, age, body height, and body weight, based on the method developed by Harris et al. [71] and adapted by Roza and Shizgal [72]. Additionally, a low energy intake cut-off value was introduced to exclude participants reporting energy intakes lower than 500 kcal/day. This procedure was presented in detail in our previous paper [73]. After the exclusion of 97 subjects (incomplete or missing anthropometric data: *n* = 12; missing one set of the 24 h recall data: *n* = 36; under-/over-reporting: *n* = 49), our study sample included *n* = 1248 subjects: 468 adolescents, 364 adults, and 416 elderly people.

To calculate the usual daily dietary vitamin B12 intake, two 24 h recalls and FPQs were used. Day-to-day inter- and intraindividual variations in the vitamin B12 intake distribution within age groups were modeled using the Multiple Source Method (MSM) [67], with participants’ age, sex, and BMI used as covariates. The MSM modeling approach using FPQ data corrects for within-individual variation in vitamin B12 intake and provides data on usual dietary intake on an individual level [74].

To assure national representativeness, the descriptive analysis considered weighting for each of the three cohorts (age/sex) using the iterative proportional fitting method [75]. Census data from the year 2017 were used for population weighting [54]. For the assessment of the proportion of each population meeting recommended daily intake, we used the nationally accepted threshold of 4 µg/day vitamin B12 [40]. For the reporting of the relative contributions of different food categories in daily vitamin B12 intake, foods were also categorized using a modified categorization system, developed by the Global Food Monitoring Initiative [76]. Population-weighted serum vitamin B12 and homocysteine levels were also calculated for all age groups and sexes. The population-weighted proportions of low vitamin B12 status and high homocysteine were calculated using the above-mentioned cut-off levels. Prevalence of risk for vitamin B12 deficiency was determined using a combination of two criteria: serum vitamin B12 concentration <148 pmol/L and homocysteine concentration >15 μmol/L.

Multiple linear and logistic regression analyses were performed to assess mean usual vitamin B12 intake and odds ratios (ORs) for inadequate daily vitamin B12 intake (<4 µg/day) in adolescents, adults, and the elderly and for selected subpopulations within these three age cohorts. In the dietary intake model analysis, we used sex, residential area, financial status, education level, BMI, IPAQ, employment status, smoking status, and following a specific medical or behavioral diet as predictor variables. Logistic regression analysis was also used to investigate risk for vitamin B12 deficiency; analysis was performed on a combined sample of adult and elderly participants (*n* = 271); 9 participants were excluded due to incomplete vitamin B12 intake data. Analyses were conducted using the following factors: age cohort, sex, residential area, financial status, smoking status, BMI, IPAQ, usual daily vitamin B12 intake, and energy intake. Furthermore, the same sample was used for linear and logistic regression analyses to assess adjusted serum homocysteine concentrations and ORs for high homocysteine concentrations (>15 μmol/L). In addition to the above-mentioned parameters, both models also included serum concentrations of vitamin B12 and folate.

All statistical analyses were performed using STATA (version 17.0; StataCorp LLC, College Station, TX, USA). The outcomes were statistically significant at *p* < 0.05, except in the logistic regression analysis for adequate daily dietary intake of vitamin B12, where marginal statistical significance is reported for *p* < 0.1.

## 3. Results

The sociodemographic characteristics of the SI.Menu sample are described in Table 1. The sample included a total of 1248 participants, divided into three population groups: adolescents (*n* = 468), adults (*n* = 364), and elderly (*n* = 416). About a quarter of these participants reported the use of multivitamin supplements, but specific data about supplementation with vitamin B12 were not available. Altogether, 34% of adults and 37% of elderly people from the SI.Menu study were further included in the Nutrihealth study. For this subsample, fasting blood samples were collected, so data on serum vitamin B12 and homocysteine levels were available and included in the analysis.

**Figure 1:**
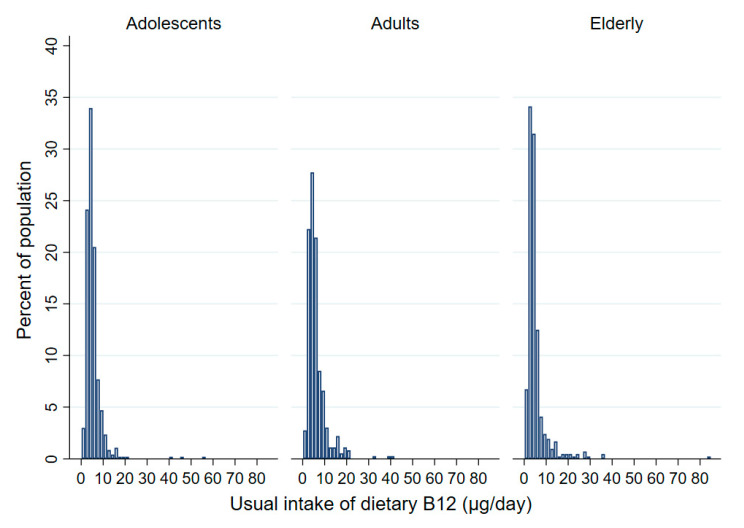
Histograms of the sample population distribution of estimated usual daily intake of vitamin B12 for different age groups (adolescents: 10–17 years; adults: 18–64 years; elderly: 65–74 years).

The estimated usual population-weighted mean daily vitamin B12 intake was above the recommended 4 µg in all age groups (Table 2). Generally, mean intake was the highest in adults (6.2 µg/day) and lower in adolescents and the elderly, where quite similar values were observed (5.4. and 5.0 µg/day, respectively). The distribution of the usual daily dietary intake of vitamin B12 among age groups is presented in Figure 1.

Despite the mean intakes being above 4 µg/day, a notable proportion of the population does not meet this threshold for recommended vitamin B12 intake (Table 2). This proportion is particularly high in the elderlies, where the recommended daily vitamin B12 intake is not met in 58.3% of the population. In adolescents and adults, the proportions of the population with inadequate vitamin B12 intakes are 37.3% and 31.7%, respectively. Lower intakes of vitamin B12 were observed in females, particularly among adolescents and adults. This trend was confirmed in the linear regression analysis, where sex was the only parameter with significant association with vitamin B12 intake (Supplementary Table S1). Interestingly, the population-weighted daily vitamin B12 intake, calculated per 1000 kcal, was also lower in females in the adult and elderly populations, but not in adolescents.

Logistic regression analysis was used to determine predictors for the prevalence of inadequate daily vitamin B12 intake below 4 µg (Table 3). This analysis also showed significantly higher odds ratios (ORs) for inadequate vitamin B12 intakes in female adolescents (OR 2.70; CI: 1.80–4.03; *p* < 0.001) and adults (OR 2.51; CI: 1.45–4.36, *p* < 0.01). While a similar trend was observed in elderly women, the difference was not significant. Particularly in adolescents and the elderly, we also observed a significant association with physical activity IPAQ score. Interestingly, a higher level of physical activity was associated with a higher prevalence of inadequate vitamin B12 intake. Marginally significant associations were found for employment (adults) and for smoking status and financial status (elderly). Compared to employed participants, those who were retired had a higher risk for inadequate daily vitamin B12 intake (OR = 2.80; CI = 1.32–5.94, *p* < 0.1). We also observed a trend of lower risk for inadequate vitamin B12 intake (OR = 0.65; CI = 0.40–1.08, *p* < 0.1) in elderly people with above-average financial status.

**Figure 2:**
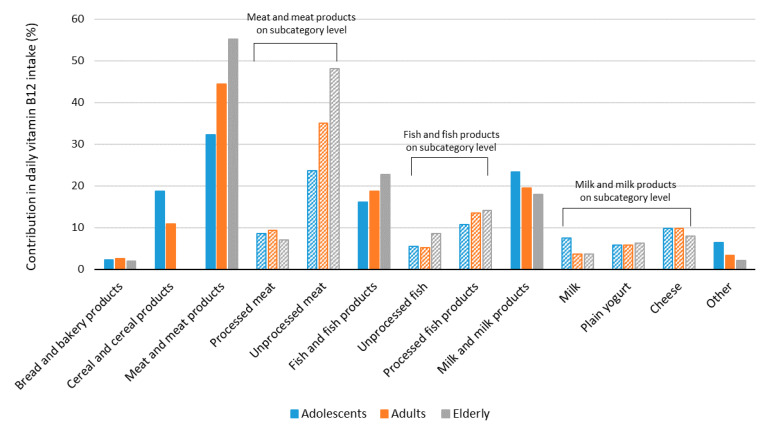
Relative contributions of selected food categories to usual daily vitamin B12 intake among different age groups (% of total vitamin B12 intake).

The relative contributions of different food categories to daily vitamin B12 intake are presented in Figure 2 and Supplementary Table S2. The most important vitamin B12 contributors were meat and meat products (including both unprocessed and processed meat), fish and fish products, and milk and milk products. In meat and meat products, which made the highest vitamin B12 contributions among all food categories in all age groups, unprocessed meat made the greatest contribution in the elderly population. In this food category, the most notable differences were observed between adolescents and the elderly, where the contribution was 32% vs. 55%, respectively. In adolescents, an important contributor to vitamin B12 intake was milk and milk products (23.4%); interestingly, another important contributor in adolescents was cereals and cereal products (18.7%), which on the other hand did not make any contribution in the elderly.

**Figure 3:**
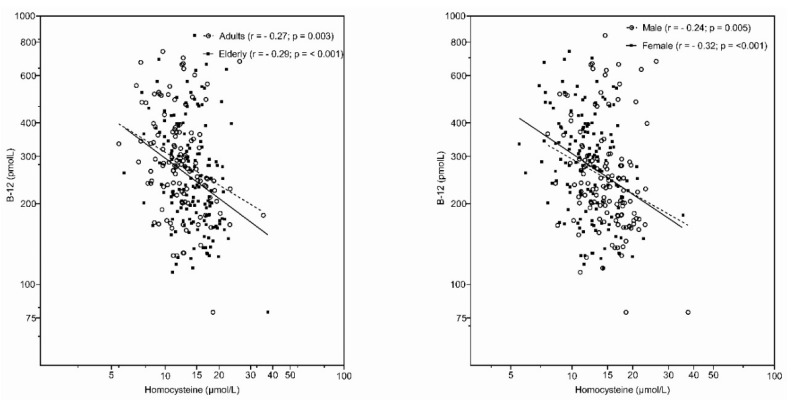
Association of serum vitamin B12 with serum homocysteine concentration in different age cohorts (**left**) and sex groups (**right**) using log scale.

As an extension of the SI.Menu study, a subsample of adults and elderly also participated in the Nutrihealth study, where blood samples were collected. Vitamin B12 status was determined using two biomarkers—serum vitamin B12 and homocysteine concentrations. The mean population-weighted serum vitamin B12 levels were 322.1 pmol/L in adults and 287.3 pmol/L in the elderly (Table 2), which are above the cut-off point for low vitamin B12 status, at 221 pmol/L. However, a low vitamin B12 status was observed in 21% of adults and in almost half of the elderly population (46%). Vitamin B12 deficiency (<148 pmol/L) was detected in 3.7% of adults and 10.4% of the elderly. The highest prevalence of low vitamin B12 status and deficiency was observed in elderly males (57.9% and 11.8%, respectively). Furthermore, when vitamin B12 deficiency was assessed together with high homocysteine (serum B12 < 148 nmol/L, serum homocysteine > 15 µmol/L), deficiency was observed in 1.2% of adults (2.3% of males and no women) and 7.0% of elderly people (7.9% of males and 6.3% of women). We should note a strong negative linear correlation between log-transformed serum vitamin B12 and homocysteine concentration in adults (r = −0.27, *p* = 0.003) and the elderly (r = −0.29, *p* < 0.001), as well as in males (r = −0.24, *p* = 0.005) and females (r = −0.32, *p* < 0.001) (Figure 3). It should be noted that serum homocysteine concentration is also affected by several other factors, including folate status. Results of the linear regression analysis model (Supplementary Table S3) identified age, sex, serum vitamin B12, and serum folate as significant parameters associated with serum homocysteine concentrations. In additional logistic regression analyses, the same parameters were also found as significant predictors of high homocysteine concentrations (>15 μmol/L): age (*p* < 0.01; higher prevalence in elderly: OR 2.42; CI: 1.23–4.73), sex (*p* < 0.001; lower prevalence in females: OR 0.21; CI: 0.1–0.41), low serum vitamin B12 concentration (*p* < 0.05; lower prevalence in those above 221 pmol/L: OR 0.52; CI: 0.28–0.99), and low serum folate (*p* < 0.01; lower prevalence in those above 7 nmol/L: OR 0.33; CI: 0.15–0.73).

**Figure 4:**
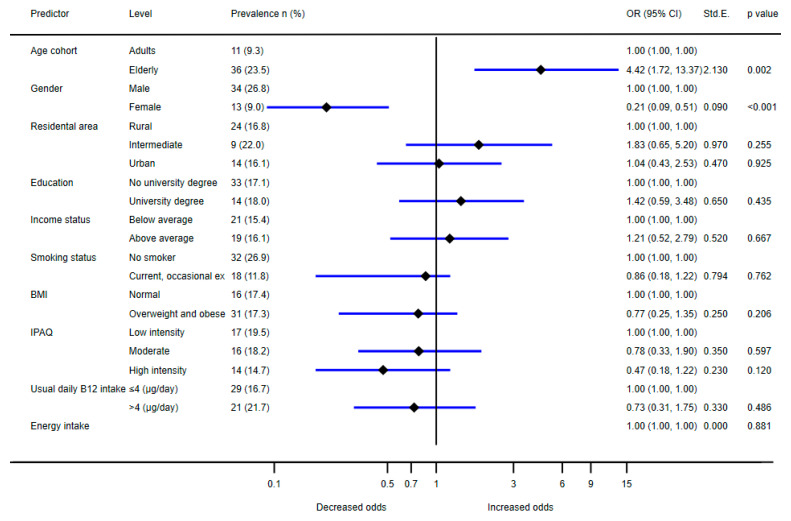
Association between prevalence of vitamin B12 deficiency (serum vitamin B12 concentration < 221 pmol/L and serum homocysteine > 15 µmol/L) and age, sex, residential area, education, financial status, body mass index (BMI), IPAQ (International Physical Activity Questionnaire) score, usual intake of vitamin B12, and usual daily energy intake.

The results of logistic regression analyses on the combined sample of adults and the elderly identified age group and sex as the only significant predictors of vitamin B12 deficiency (Figure 4). A significantly higher risk for deficiency was observed in the elderly (OR: 4.42; CI: 1.72–13.37), and lower risk was observed in females (OR: 0.24; CI: 0.09–0.51). Additionally, we tested a model which considered the use of multivitamin food supplements (in addition to the above-mentioned parameters), but this was not found as a significant predictor for vitamin B12 deficiency (*p* = 0.49).

**Figure 5:**
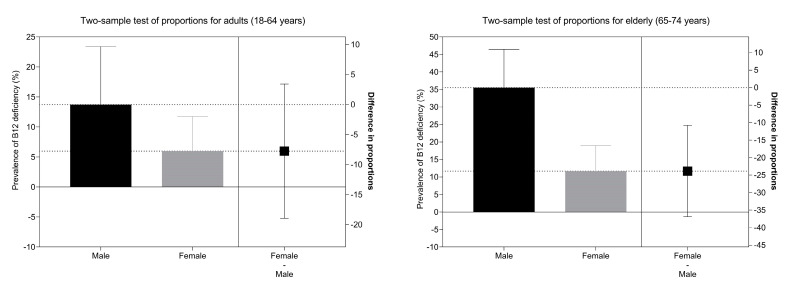
Analysis of differences in prevalence of vitamin B12 deficiency (serum vitamin B12 concentration < 221 pmol/L and homocysteine > 15 µmol/L) between males and females in different age cohorts.

Analyses of differences in the proportion of the prevalence of vitamin B12 deficiency showed significantly higher deficiency prevalence in males in the elderly population (*p* < 0.001) (Figure 5).

## 4. Discussion

Although clinically manifested vitamin B12 deficiency is relatively rare in developed countries, low intakes and statuses of vitamin B12 are much more common and can pose a public health concern, with certain population groups being particularly at risk [34]. The present study is the first one evaluating the intake and status of vitamin B12 in a nationally representative sample in Slovenia. In all population groups, the population-weighted mean vitamin B12 intakes are above the national recommendations of 4 µg/day, making the situation in Slovenia comparable to the findings from other EU countries [43,77]. In all population groups, males had higher population-weighted mean vitamin B12 intakes than women. The highest population-weighted vitamin B12 mean intake was found in the adult male population (6.9 µg/day), and the lowest was found in elderly females (4.4 µg/day). In adults and the elderly, lower mean vitamin B12 intakes in females were also found when calculating the daily vitamin B12 intake per 1000 kcal/day, while male and female adolescents had equal daily vitamin B12 intakes per 1000 kcal/day.

Even though population-weighted mean vitamin B12 intakes were generally sufficient in all age groups, more than 30% of the adolescent and adult populations did not meet the recommended daily intake of vitamin B12. Among all age groups, the situation was particularly concerning among the elderly, where 58.3% had inadequate vitamin B12 daily intakes. Looking into the distribution of vitamin B12 intake (Figure 1) especially in the elderly population, besides certain individuals with particularly low vitamin B12 intakes, there is a proportion of individuals with considerably higher vitamin B12 intakes. This influences the overall mean intake in the population, which appears higher due to the higher interindividual variability, although a notable proportion of the population did not meet the recommended daily vitamin B12 intake. Lower vitamin B12 intakes in females could be concerning, as especially in adolescents and elderly females, the median population-weighted vitamin B12 intake was lower than 4 µg/day, while consistent evidence shows that vitamin B12 intake above 4 µg/day is associated with an adequate serum vitamin B12 status [43].

Our findings are in line with the results of studies conducted in other European countries, where the reported mean intakes were usually above the national recommendations, but with notable variations across countries. In a review of studies from 15 European countries, the highest vitamin B12 intakes were seen in Finland (>7 µg/day), followed by Sweden, Germany, France, and Spain, with mean vitamin B12 intakes above 5 µg/day [48]. Denmark, Ireland, and the Netherlands had moderate vitamin intakes between 2.5 and 5 µg/day. The lowest daily vitamin B12 intake was observed in Greece (<2.5 µg/day), which is also below their national recommendation for vitamin B12 intake [78].

Dietary intakes of vitamin B12 are highly dependent on the type of diet. Diets that are low in meat and foods of animal origin are often low in vitamin B12; therefore, in countries where more foods of animal origin are consumed, vitamin B12 intakes are usually higher [1]. According to the latest study on dietary habits in Slovenia, meat is usually consumed several times weekly in all age groups, with adult males being the largest consumers of meat [79]. This is reflected in the mean vitamin B12 intakes in our study, which were also the highest in this population. As seen in Figure 2, where the relative contributions of food categories to the usual daily intake of vitamin B12 among different age groups are presented, the highest contribution of daily vitamin B12 comes from meat and meat products, with the elderly deriving notably higher levels from unprocessed meat, compared to adolescents. For adolescents, important vitamin B12 contributors were milk and milk products and, interestingly, cereal and cereal products, which made no dietary vitamin B12 contribution among the elderly. This is because fortified cereal products (i.e., breakfast cereals) can be a source of vitamin B12, but such products are more popular among adolescents than in the elderly population. We should mention that in our previous study, this food category was also found to be an important contributor to folate intake in adolescents [68]. To derive the optimal benefits from the daily diet, adolescents should be encouraged to eat more unprocessed and minimally processed foods. Although they tend to intake certain micronutrients from fortified products, such as fortified breakfast cereals, the frequent consumption of such processed foods can have detrimental effects on health. Processed foods, particularly processed breakfast cereals, can often have a less favorable nutritional composition [80] and can be high in sugar and refined grains; this was particularly observed in foods specifically marketed to children [81,82]. Considering the fact that the majority of the general population meets the daily vitamin B12 requirement, and that meats and foods of animal origin are abundantly consumed in Slovenia [79], additional encouragement of the consumption of such foods is not relevant. Currently, around 20% of daily vitamin B12 intake originates in processed meat and fish products; therefore, switching to unprocessed meat and fish should be supported.

A logistic regression analysis of adequate daily dietary intake of vitamin B12 (Table 3) showed that sex, IPAQ, smoking status, employment, and financial status were (marginally) significant predictors of adequate vitamin B12 intake, exposing adult and adolescent females, elderly nonsmokers, retired adults, elderly people with poor financial status, and all of those with higher IPAQ scores as more susceptible to inadequate daily vitamin B12 intake. This could be linked to the lower consumption of meat in females and those with lower financial status, which was also reported in the latest Slovenian dietary survey [79]. It is established that meat consumption is associated with economic status [83], and those with poor financial status usually consume less meat, especially unprocessed meat, probably also due to its higher price compared to some other foods. It was previously reported that financial status impacts food choices, which further manifests in micronutrient intake [84,85]. Regarding IPAQ score, it is possible that those who are more physically active will more commonly follow dietary restrictions, which could result in lower vitamin B12 intake. It should be mentioned that increased physical activity may raise homocysteine levels; therefore, such individuals could benefit from a higher intake of B vitamins, including vitamin B12, so as to balance homocysteine levels and reduce the risk of cardiovascular disease later in life [86].

The investigation of serum biomarkers in adults and the elderly showed that in both population groups, the weighted mean serum vitamin B12 levels were above our cut-off point for low vitamin B12 status (221 pmol/L), which is comparable to other European countries [48]. The serum vitamin B12 status was adequate in 79% of adults and 54% of the elderly. Serum vitamin B12 levels were lower in the elderly than in adults, with a higher prevalence of vitamin B12 deficiency observed in this population group, compared to adults. Higher homocysteine levels were observed in the elderly and in males, in line with previous studies [87]. The prevalence of vitamin B12 deficiency was also higher in men than in women, especially in the elderly, which is consistent with previous reports [30,88]. It is well established that certain vitamins, including vitamin B12, influence serum homocysteine status and that increased homocysteine is related to a higher risk for cardiovascular disease [89]. In the elderly population, we observed a strong negative linear association between high homocysteine and low serum vitamin B12 within age groups (Figure 3). Taking into account both low serum vitamin B12 (<148 nmol/L) and high serum homocysteine (>15 µmol/L), vitamin B12 deficiency was observed in 7% of the Slovenian elderly population, which is similar to the rates observed in the United States and the United Kingdom [90]. A much lower prevalence of deficiency (1.2%) was observed in adults. The logistic regression analysis of the prevalence of vitamin B12 deficiency (Figure 4) confirmed that elderly people and males had a higher likelihood of deficiency, while other demographic factors were not shown to be statistically significant.

Vitamin B12 intake and status are not always associated, particularly due to different causes of vitamin B12 absorption; moreover, because vitamin B12 is one of the vitamins that are best stored in the body, it can take a few years for an inadequate status to develop. This was observed, for example, in the Netherlands and Germany, where mean vitamin B12 intakes were above the recommendations, but status appeared to be inadequate [48]. We identified the elderly as a particularly vulnerable population in terms of vitamin B12 intake and status; this problem was highlighted also in numerous studies in other regions [25,91,92,93,94,95,96,97,98,99]. Depending on the diagnostic method and cut-offs, international deficiency rates among the elderly population were reported somewhere between 6% and 43%, with higher deficiency prevalence observed in developing countries [26,90,100,101]. In the elderly, the predominant factor for a high prevalence of low vitamin B12 status is malabsorption. Due to the complex absorption process of vitamin B12, several factors contribute to malabsorption, such as gastric abnormalities, small bowel diseases, pancreatic insufficiency, and several medications, most commonly protein pump inhibitors and metformin; as the age advances, the probability of such conditions increases, together with malabsorption [102]. Atrophic gastritis, often accompanied by *H. pylori* infection, is one of the more common causes of vitamin B12 deficiency, which affects 20–50% of the elderly and is typically asymptomatic [103,104]. Here, reduced gastric acid and pepsinogen secretion results in a decreased intestinal absorption of vitamin B12–protein complexes from food. Considering the high probability of malabsorption, along with the fact that 58.3% of this population had inadequate vitamin B12 intakes, this would explain our findings related to low vitamin B12 status in the elderly, as the mean population vitamin B12 intakes did not significantly deviate in comparison with other population groups. Although elderly males had higher daily vitamin B12 intakes compared to women, they also had a higher prevalence of vitamin B12 deficiency. This is seen also in Figure 5, where the data show a significantly higher deficiency prevalence in elderly males in comparison to females, while this difference was not significant for adults. As the literature’s data show that atrophic gastritis is more common in men and in the elderly [105,106,107], we could speculate that absorption impairment could be the reason for the higher vitamin B12 deficiency rates among the Slovenian elderly, especially males. The capacity for the efficient absorption of vitamin B12 from foods decreases with age; however, evidence shows that synthetic vitamin B12 from fortified foods and supplements could be more efficiently absorbed, as the synthetic form of vitamin B12 is not usually protein-bound [34]. Due to the considerable prevalence of atrophic gastritis and lower stomach acid excretion in the elderly, in some countries, certain institutions already propose that the elderly meet their daily requirements with vitamin B12 supplements and/or fortified foods [25]. Furthermore, research data show a good metabolic response to vitamin B12 supplementation in elderly people with malabsorption issues [103].

In Slovenia, testing for vitamin B12 status is currently mostly indicated for those with clinically relevant symptoms of vitamin B12 deficiency. In view of the higher prevalence of inadequate vitamin B12 intake and deficiency, as well as vitamin B12 absorption issues, in the elderly population, routine screening may be advantageous in this population. The use of vitamin B12 supplements in the elderly should also be considered in Slovenia, as the additional intake of foods naturally rich in vitamin B12 would probably not be sufficient due to absorption issues [91]. For example, because of the specific metabolic characteristics of the elderly, the American dietary guidelines have already been modified for people over 70 years of age. Dietary supplementation with vitamins B12 and D and calcium was recommended, as was the use of folic acid fortified foods, such as flour, which has been subjected to mandatory folic acid fortification in the USA since 1998 [108]. In addition, some studies suggest that besides fortifying flour with folic acid, vitamin B12 should also be added, due to the metabolic associations of these two vitamins and related public health issues [109,110]. Interventions in the elderly population are therefore much needed, as the timely detection of vitamin B12 deficiency and appropriate diagnosis could prevent severe hematological and irreversible neurological complications [111,112].

Another critical population group, for which vitamin B12 supplementation is already advised, includes those who do not consume foods of animal origin, such as vegans. In the present study, suboptimal intakes of vitamin B12 were observed for those following such diets (Supplementary Table S1), although this should be considered with caution due to the small sample size. Crude odds ratios (Table 3) also showed a higher risk for vitamin B12 deficiency in this population. Vitamin B12 intake was observed as critical in a recent systematic review of the adequacy of a vegan diet [21]. Default vitamin B12 supplementation should therefore be additionally encouraged in such individuals, and regular monitoring of vitamin B12 status should be advised.

A major strength of the present SI.Menu study was that it was performed using an internationally acknowledged EU Menu methodology for dietary intake assessment, published by the EFSA [53]. We were able to analyze nationally representative data on food consumption, using 24 h dietary recalls and FPQ. Data were collected for a representative sample of Slovenian adolescents, adults, and elderly. In a Nutrihealth study, we also investigated serum biomarkers for vitamin B12 deficiency in a subsample of adults and elderly. One of the study’s strengths was that, besides data on serum vitamin B12 concentration, the homocysteine status was also available, which supported the assessment of vitamin B12 deficiency prevalence. However, the study results should be interpreted with consideration of some limitations. The SI.Menu study only included data on the general use of specific food supplements, for example, multivitamins, but not specifically vitamin B12. Therefore, the dietary intake of vitamin B12 was only calculated from food sources. Furthermore, while a combination of serum vitamin B12 and homocysteine concentration is considered a much better indicator for the assessment of vitamin B12 deficiency than serum vitamin B12 alone, we were not able to analyze methylmalonic acid, which is also considered a very valuable biomarker for the assessment of vitamin B12 status [33]. We should also note that adults in the Nutrihealth study were slightly overrepresented by those above 50 years of age. Another limitation is that serum biomarker data were not available for adolescents. Furthermore, although the standard chemiluminescence immunoassay medical diagnostic method was used to determine vitamin B12 in serum samples, we should note that this method is sensitive to very high biotin concentrations. In serum samples with biotin concentration above 1500 µg/L, the measurement error was less than 10%, and therefore serum biotin concentration was not controlled.

## 5. Conclusions

In Slovenia, the estimated population-weighted mean daily vitamin B12 intake was above the recommended 4 µg in all age groups. However, inadequate daily vitamin B12 intake was observed in 37.3% of adolescents, 31.7% of adults, and 58.3% of elderly. The mean population-weighted daily vitamin B12 intake was lower in females in all population groups. Besides sex, other notable predictors for inadequate vitamin B12 intake were physical activity IPAQ score, financial status, and smoking and employment status. The study also showed that in all population groups, the most important contributors to vitamin B12 intake were meat and meat products, followed by milk and milk products. The mean population-weighted serum vitamin B12 concentration was 322.1 pmol/L in adults and 287.3 pmol/L in the elderly population. Vitamin B12 deficiency (<148 pmol/L, homocysteine >15 µmol/L) was observed in 1.2% of adults and 7.0% of the elderly. In the elderly, vitamin B12 malabsorption jeopardizes sufficient vitamin B12 status. Our study identified the male elderly population as especially at risk for low vitamin B12 status. Particularly in the elderly, monitoring for vitamin B12 is very relevant, and supplementation could be considered.

## Figures and Tables

**Table 1:** Demographic characteristics of the SI.Menu study sample for all three age cohorts (adolescents: 10–17 years; adults: 18–64 years; elderly: 65–74 years).

Variable		Adolescents *n* (%)	Adults *n* (%)	Elderly *n* (%)
Overall		*n* = 468 (100)	*n* = 364 (100)	*n* = 416 (100)
Age (mean ± SD)		13.4 (2.37)	43.6 (13.81)	68.7 (2.7)
Residential area	rural	270 (57.7)	202 (55.5)	229 (55.1)
	intermediate	76 (16.2)	56 (15.4)	71 (17.1)
	urban	122 (26.1)	106 (29.1)	116 (27.9)
Sex	male	238 (50.9)	173 (47.5)	213 (51.2)
	female	230 (49.1)	191 (52.5)	203 (48.8)
Education	no university degree	n.a.	249 (68.4)	342 (82.2)
	university degree	n.a.	115 (31.6)	74 (17.8)
Financial status	below average	n.a.	118 (38.4)	269 (71.5)
	above average	n.a.	189 (61.6)	107 (28.5)
Employment	employed	n.a.	226 (62.1)	n.a.
	unemployed	n.a.	42 (11.5)	n.a.
	student	n.a.	32 (8.8)	n.a.
	retired	n.a.	64 (17.6)	n.a.
BMI (mean ± SD)		21.0 (4.2)	26.7 (5.2)	28.4 (5.0)
BMI	normal	301 (64.6)	148 (40.7)	108 (26.0)
	overweight and obese	167 (35.7)	216 (59.3)	308 (74.0)
Smoking status	current, occasional, ex-smoker	30 (6.4)	165 (45.3)	185 (44.5)
	nonsmoker	438 (93.6)	199 (54.7)	231 (55.5)
IPAQ	low	108 (23.3)	127 (35.3)	137 (33.4)
	moderate	141 (30.5)	108 (30.0)	133 (32.4)
	high	214 (46.2)	125 (34.7)	140 (34.2)
Supplement use	multivitamins	129 (27.6)	140 (38.4)	95 (22.8)
	use not reported	339 (72.4)	224 (61.5)	321 (77.2)
Diet type	vegetarian/vegan	12 (2.6)	8 (2.2)	3 (0.7)
	no diet	456 (97.4)	356 (97.8)	413 (99.3)
	medical and/or weight loss	13 (2.8)	32 (8.8)	51 (12.3)
	no diet	455 (97.2)	332 (91.2)	465 (87.7)
Participation in the Nutrihealth study *			125 (34.3)	155 (37.3)

Notes: SD = standard deviation; BMI = body mass index; for adults and elderly people, normal BMI was considered below 25 kg/m^2^, while sex-/age-adjusted cut-off points were used for adolescents [61,62]; IPAQ = physical activity according to International Physical Activity Questionnaire; * serum vitamin B12 and homocysteine levels available for the subgroup participating in the Nutrihealth study. n.a.: not applicable.

**Table 2:** Population-weighted usual daily vitamin B12 intake and prevalence of inadequate vitamin B12 intake (<4 µg/day) and serum markers for deficiency.

	Adolescents *n* (%)			Adults *n* (%)			Elderly *n* (%)		
	All	Male	Female	All	Male	Female	All	Male	Female
SI.Menu study *n* (%)	468 (100)	238 (50.9)	230 (49.2)	364 (100)	173 (47.5)	191 (52.5)	416 (100)	213 (51.2)	203 (48.8)
Weighted * *n* (%)	150,674 (78.2)	75,580 (50.2)	73,094 (49.8)	1,302,132 (78.2)	670,464 (51.5)	631,668 (48.5)	212,793 (12.8)	100,247.5 (47.1)	112,545.5 (52.9)
Usual daily vitamin B12 intake									
Mean (95%CI) (µg/day)	5.4 (5.0–5.8)	6.0 (5.4–6.5)	4.7 (4.1–5.4)	6.2 (5.7–6.8)	6.9 (6.1–7.8)	5.5 (4.8–6.2)	5.0 (4.4–5.6)	5.7 (4.5–6.8)	4.4 (3.8–5.0)
Q25 (µg/day)	3.4	4.4	2.8	3.6	4.3	3.3	2.9	3.2	2.7
Median (µg/day)	4.7	5.3	3.9	5.0	5.4	4.4	3.6	3.8	3.4
Q75 (µg/day)	5.9	6.7	5.4	7.1	8.1	6.1	4.9	5.5	4.5
Mean (95%CI) (µg/per 1000 kcal/day)	2.0 (1.9–2.2)	2.0 (1.8–2.3)	2.0 (1.8–2.3)	2.5 (2.3–2.7)	2.5 (2.2–2.9)	2.4 (2.1–2.6)	2.1 (1.9–2.4)	2.3 (1.8–2.7)	2.0 (1.7–2.2)
Prevalence of inadequate daily vitamin B12 intake (< 4µg) (%) (95% CI)									
<4 µg/day	37.3 (30.6–44.6)	24.0 (17.7–31.8)	51.7 (42.2–61.2)	31.7 (26.5–37.3)	20.6 (14.9–27.8)	42.9 (35.1–51.0)	58.3 (49.5–66.7)	53.2 (37.7–68.0)	63.1 (52.7–72.3)
Nutrihealth study *n* (%)				125 (100)	52 (41.6)	73 (58.4)	155 (100)	76 (49.0)	79 (51.0)
Serum vitamin B12 (pmol/L) (95% CI)									
Mean (95%CI)				322.1	329.8	314.0	287.3	250.2	320.3
				(294.2–350.1)	(284.7–374.9)	(282.2–345.7)	(258.2–316.2)	(219.1–281.2)	(274.1–366.5)
Std. Err.				14.1	22.8	16.1	14.7	15.8	23.4
Median				283	280	283	232	204	232
Prevalence of low serum vitamin B12 (%) (95% CI)									
<148 pmol/L				3.7 (1.6–8.3)	2.4 (0.6–9.2)	5.3 (1.9–13.9)	10.4 (6.4–16.4)	11.8 (6.2–21.4)	9.1 (4.4–18.0)
<221 pmol/L				21.1 (14.5–29.7)	18.9 (10.2–32.2)	23.6 (14.8–35.5)	46.0 (38.2–54.1)	57.9 (46.5–68.5)	35.1 (25.2–46.4)
Serum homocysteine (µmol/L) (95% CI)									
Mean (95% CI)				12.6 (11.9–13.3)	13.6 (12.6–14.6)	14.6 (13.9–15.2)	14.6 (13.9–15.2)	16.1 (15.2–17.0)	13.2 (12.5–13.9)
Std. Err.				0.35	0.50	0.42	0.32	0.47	0.36
Median				12.1	12.6	15.7	14.2	10.9	13.0
Prevalence of high serum homocysteine (µmol/L) (%) (95% CI)									
>10 µmol/L				75.3 (66.4–82.4)	88.7 (76.5–95.0)	61.0 (48.6–72.2)	88.9 (82.7–93.0)	96.0 (88.3–98.7)	82.5 (72.5–89.4)
>15 µmol/L				20.5 (13.9–29.1)	26.4 (15.8–40.8)	14.2 (8.0–23.9)	39.9 (32.4–47.8	56.6 (45.2–67.3)	25.0 (16.7–35.7)
Prevalence of vitamin B12 deficiency (%) (95% CI) using criteria for low serum vitamin B12 (<148 nmol/L) and high serum homocysteine (>15µmol/L)									
				1.2 (0.2–4.7)	2.3 (0.5–9.1)	/	7.0 (3.9–12.3)	7.9 (3.6–16.6)	6.3 (2.6–14.3)

Notes: CI—confidence interval. Dietary intake of Vitamin B12 is estimated with consideration of regular foods (without food supplements). * Number of people and their respective population share regarding age and sex cohorts (census data in 2017). Serum homocysteine levels from [68].

**Table 3:** Association between the prevalence of inadequate intake of vitamin B12 (<4 µg/day) and sex, place of living, education, income, employment, smoking status, BMI, IPAQ, vegetarian/vegan diet, and diet restrictions for different age groups.

Variable		Adolescents (10–17 Years)			Adults (18–64 Years)			Elderly (65–74 Years)		
		Prevalence (%)	Crude OR	Adjusted OR	Prevalence (%)	Crude OR	Adjusted OR	Prevalence (%)	Crude OR	Adjusted OR
**Overall**		175 (37.4)			118 (32.4)			215 (51.7)		
Sex	male	62 (26.1)	1	1	40 (23.1)	1	1	94 (44.1)	1	1
	female	113 (49.1)	2.74 (1.83–4.12)	2.70 (1.80–4.03)	78 (40.8)	2.29 (1.42–3.73)	2.51 (1.45–4.36)	121 (59.6)	1.87 (1.24–2.81)	1.44 (0.90–2.31)
Place of living	rural	99 (36.7)	1	1	68 (33.7)	1	1	115 (50.2)	1	1
	intermediate	24 (31.6)	0.80 (0.44–1.41)	0.78 (0.44–1.38)	22 (39.3)	1.27 (0.66–2.44)	1.27 (0.63–2.59)	41 (57.8)	1.35 (0.77–2.41)	1.34 (0.74–2.43)
	urban	52 (42.6)	1.28 (0.81–2.03)	1.20 (0.76–1.92)	28 (26.4)	0.71 (0.40–1.22)	0.73 (0.40–1.33)	59 (50.9)	1.03 (0.64–1.64)	1.02 (0.61–1.70)
Education	no university degree		n.a.	n.a.	78 (31.3)	1	1	180 (52.6)	1	1
	university degree				40 (34.8)	1.17 (0.71–1.91)	1.62 (0.88–3.00)	35 (47.3)	0.81 (0.47–1.37)	0.94 (0.51–1.72)
Financial status	below average		n.a.	n.a.	40 (33.9)	1	1	148 (55.0)	1	1
	above average				57 (30.2)	0.84 (0.50–1.42)	0.94 (0.51–1.71)	48 (44.9)	0.67 (0.41–1.07)	0.65 (0.40–1.08)
BMI	normal	110 (35.5)	1	1	48 (32.4)	1	1	57 (52.8)	1	1
	overweight and obese	65 (38.9)	1.10 (0.73–1.66)	1.13 (0.75–1.72)	70 (32.4)	1.00 (0.62–1.60)	1–03 (0.59–1.80)	158 (51.3)	0.94 (0.59–1.50)	1.00 (0.61–1.65)
IPAQ	low intensity	27 (25.0)	1	1	36 (28.4)	1	1	64 (46.7)	1	1
	moderate	68 (48.2)	2.79 (1.57–5.03)	2.48 (1.41–4.38)	33 (30.6)	1.11 (0.61–2.02)	1.28 (0.66–2.46)	83 (62.4)	1.89 (1.13–3.17)	1.86 (1.10–3.14)
	high intensity	76 (35.5)	1.65 (0.96–2.89)	1.64 (0.96–2.80)	47 (37.6)	1.52 (0.87–2.68)	2.13 (1.12–4.03)	64 (45.7)	0.96 (0.58–1.58)	1.02 (0.61–1.71)
Employment	employed		n.a.	n.a.	65 (28.8)	1	1		n.a.	n.a.
	unemployed				15 (35.7)	1.37 (0.64–2.88)	1.71 (0.72–4.05)			
	student				8 (25.0)	0.83 (0.30–2.02)	1.13 (0.38–3.34)			
	retired				30 (46.9)	2.19 (1.18–4.01)	2.80 (1.32–5.94)			
Smoking status	nonsmoker	164 (37.4)	1	1	64 (32.2)	1	1	133 (57.6)	1	1
	current/ex-smoker	11 (36.7)	0.97 (0.41–2.20)	0.96 (0.42–2.20)	54 (32.7)	1.03 (0.64–1.63)	1.02 (0.59–1.76)	82 (44.3)	0.59 (0.39–0.88)	0.64 (0.40–1.02)
Medical diet	no special diet	169 (37.1)	1	1	105 (31.6)	1	1	184 (50.4)	1	1
	medical/weight loss	6 (46.2)	1.45 (0.40–5.13)	1.61 (0.49–5.26)	13 (40.6)	1.48 (0.64–3.29)	1.28 (0.53–3.08)	31 (60.8)	1.52 (0.81–2.93)	1.34 (0.70–2.56)
Behavioral diet	no diet	166 (36.4)	1	n.a.	113 (31.7)	1	n.a.	212 (51.3)	n.a.	n.a.
	vegetarian/vegan	9 (75.0)	5.24 (1.28–30.40)	n.a.	5 (62.5)	3.58 (0.68–23.39)	n.a.	3 (100.0)	n.a.	n.a.

Notes: n.a.—not applicable; CI—confidence interval; body mass index (BMI) was considered as normal below 25 kg/m^2^, except for adolescents, where sex-/age-adjusted cut-off points [61,62] were used. Logistic regression analysis was conducted on samples with excluded missing values (financial status: *n* = 57 (adults) and 40 (elderly); IPAQ (International Physical Activity Questionnaire): *n* = 5 (adolescents), 4 (adults), 6 (elderly)). Cut-off odds ratios calculated with threshold of vitamin B12 intake < 4 µg/day; association was significant (*p* < 0.05) or marginally significant (*p* < 0.1) for the following variables: *p* < 0.001 sex (adolescents), *p* < 0.01 IPAQ (adolescents); *p* < 0.01 sex (adults), *p* < 0.1 IPAQ (adults), *p* < 0.1 employment (adults); *p* < 0.05 IPAQ (elderly), *p* < 0.1 smoking status (elderly), *p* < 0.1 financial status (elderly).

## Data Availability

The data presented in this study are available on request from the corresponding author.

## Supplementary Materials

The following are available online at https://www.mdpi.com/article/10.3390/nu14020334/s1. Supplementary Table S1. Mean (SD) and model-adjusted mean (95% CI) levels of usual daily intake of vitamin B12 by sex, residential area, education, financial status, employment, vegetarian/vegan diet, smoking status, dietary restrictions, BMI, and IPAQ score for different age cohorts. Supplementary Table S2. The relative contributions of selected food categories to usual daily dietary vitamin B12 intake among different age groups (% of total dietary vitamin B12 intake). Supplementary Table S3. Mean (SD) and model-adjusted mean (95% CI) serum homocysteine concentration (µmol/L) by age, sex, residential area, education, financial status, smoking status, BMI, IPAQ, diet, use of multivitamin supplements, serum folate concentration (nmol/L), and serum vitamin B12 concentration (pmol/L).
