# Supplementary material for: Dietary Intake and Status of Vitamin B12 in Slovenian Population
Source: Nutrients. 2022 Jan 13;14(2):334. doi: 10.3390/nu14020334 (PMC8781642; doi:10.3390/nu14020334)
Supplement: Supplementary file 1 [file nutrients-14-00334-s001.zip › nutrients-1542890-supplementary-done.pdf]

nutrients

MDPI

Supplementary Table S1. Mean (SD) and model-adjusted mean (95% CI) levels of usual daily intake of vitamin B12 by sex, residential area, education, financial status, employment, vegetarian/vegan diet, smoking status, dietary restrictions, BMI, and IPAQ score for different age cohorts.

Variable	Level	Adolescents (10–17 Years Old)			Adults (18–64 Years Old)			Elderly (65–74 Years Old)		
		N	Mean	Adjusted mean	N	Mean	Adjusted mean	N	Mean	Adjusted mean
Overall N (%)		468	5.4 (4.4)		364	6.1 (4.6)		416	5.4 (6.1)	
Sex	male	238	6.0 (4.8)	6.0 (5.4–6.6)	173	6.8 (4.8)	7.0 (6.2–7.8)	213	6.1 (7.6)	5.9 (5.0–6.9)
	female	230	4.8 (3.9)	4.8 (4.3–5.4)	191	5.5 (4.4)	5.5 (4.8–6.3)	203	4.6 (3.8)	4.9 (3.9–5.8)
Residential area	rural	270	5.3 (4.2)	5.3 (4.8–5.9)	202	6.0 (3.9)	6.0 (5.3–6.7)	229	5.7 (7.1)	5.7 (3.9–5.5)
	intermediate	76	5.1 (2.4)	5.2 (4.2-6.2)	56	5.4 (3.0)	5.5 (4.2–6.9)	71	4.8 (4.7)	5.0 (3.5–6.6)
	urban	122	5.8 (5.6)	5.8 (5.0–6.6)	106	6.8 (6.3)	6.9 (6.0–7.9)	116	5.0 (4.4)	5.0 (3.8–6.2)
Education	no university	n.a.	n.a.	n.a.	249	5.9 (3.8)	6.0 (5.3–6.7)	342	5.4 (6.4)	5.4 (4.7–6.1)
	university degree				115	6.7 (6.1)	6.7 (5.7–7.8)	74	5.4 (4.4)	5.4 (3.8–7.0)
Financial status	below average	n.a.	n.a.	n.a.	118	6.4 (5.6)	6.8 (5.8-7.7)	269	5.2 (6.4)	5.2 (4.4–5.9)
	above average				189	6.1 (4.4)	5.9 (5.2-6.6)	107	5.9 (5.7)	6.0 (4.7–7.2)
Employment	employed	n.a.	n.a.	n.a.	226	6.4 (5.2)	6.6 (5.9–7.4)	n.a.	n.a.	n.a.
	unemployed				42	5.5 (3.9)	5.6 (3.9–7.2)			
	student				32	6.2 (2.8)	6.2 (4.1–8.3)			
	retired				64	5.3 (3.5)	5.2 (3.9–6.6)			
Smoking status	current, ex-smoker	30	5.5 (3.1)	5.3 (3.7-6.9)	165	6.2 (5.0)	6.4 (6.0-7.2)	185	5.9 (5.8)	5.6 (4.6-6.6)
	non smoker	438	5.4 (4.5)	5.4 (5.0-5.9)	199	6.0 (4.4)	6.1 (5.3-6.8)	231	5.0 (6.3)	5.2 (4.3-6.1)
BMI	normal	301	5.5 (4.3)	5.5 (5.0–6.0)	148	6.3 (5.1)	6.6 (5.7–7.4)	108	4.9 (4.2)	5.3 (4.0–6.6)
	overweight and	167	5.3 (4.7)	5.3 (4.6–5.9)	216	6.0 (4.3)	6.0 (5.3–6.7)	308	5.5 (6.6)	5.4 (4.7–6.2)
IPAQ	low intensity	108	5.6 (3.0)	5.6 (4.7–6.4)	127	6.1 (4.5)	6.4 (5.5–7.3)	137	5.6 (5.5)	5.6 (4.5–6.7)
	moderate	141	5.6 (6.8)	5.7 (4.9–6.4)	108	6.3 (5.3)	6.6 (5.6–7.6)	133	4.5 (3.7)	4.6 (3.5–5.7)
	high intensity	214	5.2 (2.8)	5.2 (4.6–5.8)	125	5.9 (4.1)	5.7 (4.8–6.6)	140	6.1 (8.1)	6.0 (4.9–7.1)
Dietary restrictions	no special diet	455	5.4 (4.4)	5.4 (5.0-5.8)	332	6.0 (4.5)	6.2 (5.6-6.7)	365	5.3 (4.8)	5.3 (4.6-6.0)
	medical and/or weight loss	13 (2.8)	6.1 (4.4)	6.0 (3.7-6.9)	32 (8.8)	7.2 (5.8)	7.0 (5.1-8.8)	51 (12.3	5.9 (11.6)	6.2 (4.4-8.0)
Behavioural diet	no diet	456	5.5 (4.4)	n.a.	356	6.2 (4.6)	n.a.	413	5.4 (6.1)	n.a.
	veget./vegan	12	4.4 (3.5)	n.a.	8	4.8 (5.2)	n.a.	3	2.5 (1.8)	n.a.

Note: Body mass index (BMI) was considered as normal below 25 kg/m^2, except for adolescents, where sex-/age-adjusted cut-off points [62,63] were used. Linear regression analysis conducted on samples with excluded missing values (Financial status: $n = 57$ (adults) and 40 (elderly); International Physical Activity Questionnaire (IPAQ): $n = 5$ (adolescents), 4 (adults), 6 (elderly)); difference in marginal linear predictions per different socio-demographic and other individual related characteristics within age categories: $p < 0.01$ sex (adolescents); $p = 0.01$ sex (adults).

Supplementary Table S2. The relative contributions of selected food categories to usual daily dietary vitamin B12 intakes among different age groups (% of total dietary vitamin B12 intake)

Food category	Adolescents (10-17)	Adults (18-64)	Elderly (65-74)
**Bread and bakery products**	**2.2**	**2.7**	**1.9**
Bread	0.7	0.4	0.2
Biscuits	0.6	0.4	0.4
Cakes, muffins, and pastry	1.0	2.0	1.4
**Cereal and cereal products**	**18.7**	**10.9**	**0.0**
**Convenience foods**	0.7	0.4	0.1
**Meat and meat products**	**32.3**	**44.5**	**55.2**
Processed meat	8.6	9.4	7.1

Unprocessed meat	23.7	35.1	48.1
**Fish and fish products**	**16.2**	**18.8**	**22.8**
Unprocessed fish	5.5	5.2	8.6
Processed fish products	10.7	13.6	14.2
**Milk and milk products**	**23.4**	**19.5**	**18.0**
Milk	7.5	3.6	3.6
Plain yogurt	5.9	5.9	6.2
Cheese	9.9	9.9	7.9
**Other**	**6.5**	**3.4**	**2.1**

Supplementary Table S3. Mean (SD) and model-adjusted mean (95% CI) serum homocysteine concentration (μmol/L) by age, sex, residential area, education, financial status, smoking status, BMI, IPAQ, diet, use of multivitamin supplements, serum folate concentration (nmol/L) and serum vitamin B12 concentration (pmol/L).

Variable	Level	N (%)	Mean (SD)	Adjusted mean (CI)
Overall N (%)		271 (100)	13.8 (4.2)	
Age	adults	118 (43.5)	12.7 (4.1)	12.7 (12.0–13.5)
	elderly	153 (56.5)	14.6 (4.0)	14.3 (13.7–14.9)
Sex	male	127 (46.9)	15.2 (4.2)	15.1 (14.3–15.8)
	female	144 (53.1)	12.5 (3.7)	11.7 (11.7–13.1)
Residential area	rural	143 (52.8)	14.2 (4.7)	13.9 (13.2–14.6)
	intermediate	41 (15.1)	13.5 (3.8)	13.5 (12.3-14.6)
	urban	87 (32.1)	13.2 (3.3)	13.3 (12.5–14.1)
Education	no university degree	193 (71.2)	14.4 (4.3)	13.8 (13.2–14.4)
	university degree	78 (28.8)	13.1 (3.6)	13.2 (14.2–14.1)
Financial status	below average	136 (53.5)	14.0 (3.8)	13.7 (13.0–14.4)
	above average	118 (46.5)	13.2 (4.4)	13.5 (12.8–14.3)
Smoking status	current, ex-smoker	119 (43.9)	13.4 (4.2)	13.8 (13.1-14.5)
	non smoker	152 (56.1)	14.2 (4.1)	13.5 (12.8-14.1)
BMI	normal	92 (34.0)	13.4(5.0)	13.7 (12.9–14.6)
	overweight and obese	179 (66.0)	14.0 (3.6)	13.6 (13.0–14.1)
IPAQ	low intensity	87 (32.2)	13.5 (4.0)	13.5 (12.6–14.3)
	moderate	88 (32.6)	14.1 (4.3)	14.1 (13.2–14.8)
	high intensity	95 (35.2)	13.7 (4.3)	13.3 (12.5–14.1)
Special diet	no special diet	245 (90.4)	13.7 (4.0)	13.6 (13.1-14.1)
	medical and/or weight loss	26 (9.6)	14.2 (5.6)	14.2 (12.7-15.6)
Behavioural diet	no diet	267 (98.5)	13.7 (3.9)	13.6 (13.1–14.0)
	vegetarian/vegan	4 (1.5)	17.9 (13.1)	18.3 (14.4–22.1)
Use of multi-vitamin supplements	not supplementing	169 (62.4)	14.1 (4.4)	13.5 (12.8–14.1)
	supplementing	102 (37.6)	13.2 (3.7)	13.9 (12.7–15.6)

Note: Body mass index (BMI) was considered as normal below 25 kg/m^2. Analysis conducted on samples with excluded missing values (financial status, n=17; International Physical Activity Questionnaire (IPAQ), n=1; education, n=1; employment, n=1)). Significant association was detected for the following predictors: age ($p<0.01$), sex ($p<0.001$), behavioural diet ($p<0.05$), serum folate ($\beta = -0.06$, $p<0.05$), serum vitamin B12 ($\beta = -0.004$, $p<0.05$). Difference in marginal linear predictors per different socio-demographic and other categorical predictors: $p < 0.01$ age; $p < 0.01$ sex; $p<0.05$ behavioural diet.
